# The RTM Resistance to Potyviruses in *Arabidopsis thaliana*: Natural Variation of the *RTM* Genes and Evidence for the Implication of Additional Genes

**DOI:** 10.1371/journal.pone.0039169

**Published:** 2012-06-18

**Authors:** Patrick Cosson, Valérie Schurdi-Levraud, Quang Hien Le, Ophélie Sicard, Mélodie Caballero, Fabrice Roux, Olivier Le Gall, Thierry Candresse, Frédéric Revers

**Affiliations:** 1 INRA, UMR 1332 de Biologie du fruit et Pathologie, Villenave d’Ornon, France; 2 Univ. Bordeaux, UMR 1332 de Biologie du fruit et Pathologie, Villenave d’Ornon, France; 3 FRE CNRS 3268 – Laboratoire de Génétique et Evolution des Populations Végétales, Université des Sciences et Technologies de Lille 1, Villeneuve d’Ascq, France; Friedrich-Alexander-University Erlangen-Nurenberg, Germany

## Abstract

**Background:**

The non conventional RTM (Restricted *Tobacco etch virus* Movement) resistance which restricts long distance movement of some plant viruses in *Arabidopsis thaliana* is still poorly understood. Though at least three *RTM* genes have been identified, their precise role(s) in the process as well as whether other genes are involved needs to be elucidated.

**Methodology/Principal Findings:**

In this study, the natural variation of the *RTM* genes was analysed at the amino acid level in relation with their functionality to restrict the long distance movement of *Lettuce mosaic potyvirus* (LMV). We identified non-functional *RTM* alleles in LMV-susceptible Arabidopsis accessions as well as some of the mutations leading to the non-functionality of the RTM proteins. Our data also indicate that more than 40% of the resistant accessions to LMV are controlled by the RTM genes. In addition, two new *RTM* loci were genetically identified.

**Conclusions/Significance:**

Our results show that the RTM resistance seems to be a complex biological process which would involves at least five different proteins. The next challenges will be to understand how the different RTM protein domains are involved in the resistance mechanism and to characterise the new RTM genes for a better understanding of the blocking of the long distance transport of plant viruses.

## Introduction

Systemic infection of plants by viruses is the result of compatible interactions between plant and viral factors. These molecular interactions control translation and replication of the viral nucleic acid(s) and generalized invasion of the host through cell-to-cell and long distance movements of viral particles or ribonucleoprotein complexes [1], [2]. Plants have developed various mechanisms of resistance against viruses. Passive resistances generally result in incompatible interactions of plant and viral factors, blocking the viral cycle step(s) in which the particular interaction is involved, and are usually controlled by recessive resistance genes [3]. Active resistances are generally triggered by the recognition of viral factors by plant sensors and are controlled by at least two types of mechanisms. One well known mechanism is associated with the hypersensitive response (HR) or extreme resistance at initial infection sites and is controlled by dominant resistance R genes through a gene-for-gene relationship [4], [5]. The second mechanism concerns the general antiviral defence system of RNA interference, which targets the viral nucleic acids [5], [6].

The RTM resistance genes are atypical R genes which restrict the long distance movement of several potyviruses in *Arabidopsis thaliana* [7], [8]. In this resistance process, viral replication and cell-to-cell movement in inoculated leaves appear unaffected, HR and systemic acquired resistance are not triggered and salicylic acid is not involved [7]. First thought to be specific to *Tobacco etch virus* (TEV), this resistance was later shown to be active against at least two other potyviruses, *Lettuce mosaic virus* (LMV) and *Plum pox virus* (PPV) [8], [9]. Genetic characterization of natural Arabidopsis accession variation and of chemically induced mutants revealed that at least three dominant genes, named *RTM1*, *RTM2* and *RTM3* (for Restricted TEV Movement) [7], [10], are involved in this resistance. A single mutation in one of the *RTM* genes is sufficient to abolish the resistance phenotype [10]. *RTM1* (At1g05760) encodes a protein belonging to the jacalin family some members of which are involved in defence against insects and fungi [11]. *RTM2* (At5g04890) encodes a protein with similarities to small heat shock proteins and containing a transmembrane domain [12]. Expression of *RTM2* is however not heat inducible and does not contribute to thermo-tolerance. Both *RTM1* and *RTM2* are expressed in phloem-associated tissues and the corresponding proteins localize to sieve elements [13]. *RTM3* (At3g58350) has been recently cloned and encodes a protein belonging to an undescribed protein family that has a meprin and TRAF homology (MATH) domain in its amino-terminal region and a coiled-coil domain at its carboxy-terminal end and which interacts with RTM1 [14]. None of the RTM proteins has been found to interact with the coat protein (CP) of potyviruses [14], despite the fact that the CP harbours the viral determinant involved in the overcoming of the RTM resistance [15]. Overall, the molecular mechanisms underlying the RTM resistance are still far from understood.

In an effort to participate in the elucidation of this original resistance mechanism, we undertook the study of the natural genetic diversity of the *RTM* genes in relation with their resistance function. The present study addresses two main questions: (i) what is the basis of the inactivation of the RTM resistance in LMV-susceptible Arabidopsis accessions? And (ii) are there other RTM genes involved in the resistance process?

## Results

### Natural Genetic Variation of the *RTM* Genes among Arabidopsis Accessions

To explore the natural diversity of the *RTM* genes, genomic DNA sequencing of the coding regions (excluding the 5′ and 3′ UTR but including introns) of the *RTM1, RTM2* and *RTM3* genes from a set of 31 Arabidopsis accessions covering a large genetic diversity (Table S1, [16]) was performed. The sequences obtained were compared with the reference complete Col-0 genome sequence. All three genes could be amplified and sequenced in all accessions tested. The polymorphisms and the diversity identified at the nucleotide level in each gene are summarised in Figure S1 and Table S2. The nucleotide diversity (π, which does include insertion-deletion polymorphisms) appears higher for *RTM3* than for *RTM1* and *RTM2* with contrasting patterns among the *RTM* genes when comparing nucleotide diversity between coding and noncoding regions (Table S2). The coding region of *RTM1* was less diverse (π = 0.0037±0.0013) than its noncoding regions (π = 0.0107±0.0068), while the coding region of *RTM2* was more diverse (π = 0.0023±0.0007) than its noncoding regions (π = 0.0009±0.0008). The coding region of *RTM3* was as diverse (π = 0.0112±0.0021) as its non coding regions (π = 0.0165±0.0049).

**Figure 1:**
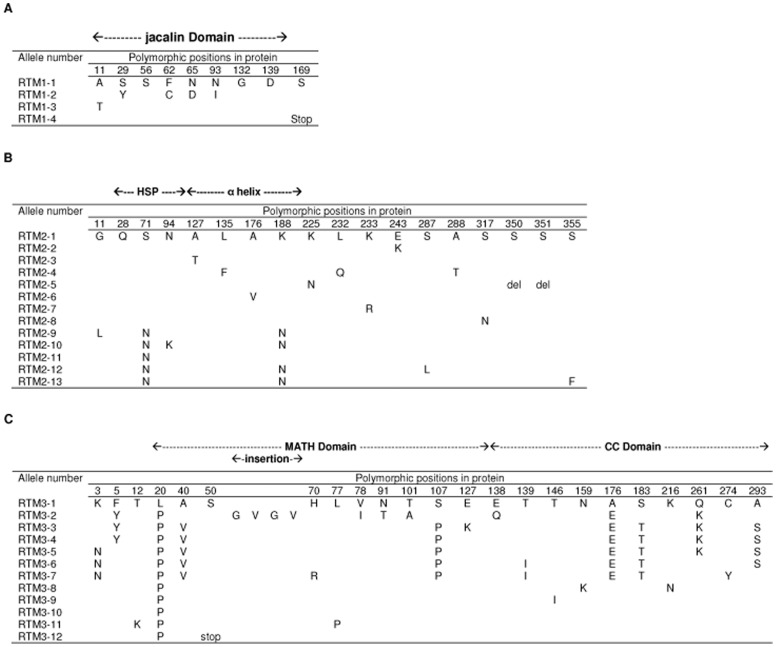
Amino acid changes in the different allelic forms of the three RTM proteins. Numbers in the first line correspond to the position of the amino acid changes in each RTM protein according to the Col-0 sequence which corresponds to the allele number 1. The different protein domains are delimited by arrows above the table. (**A**) Amino acid changes in RTM1; (**B**) Amino acid changes in RTM2; (**C**) Amino acid changes in RTM3.

At the protein level, 3, 12 and 11 different protein sequences were identified in addition to the Col-0 one for RTM1, RTM2 and RTM3 respectively (Fig.1). Only one supplementary predicted amino acid sequence was found in RTM1 (RTM1-3) as the RTM1-2 sequence found in Bl-1 and Ct-1 and the RTM1-4 sequence found in Ler-2 were previously identified in the C24 and La-er accessions respectively [11]. In this new RTM1 form, a threonine instead of an alanine at position 11 was found in Kn-0. In RTM1-2 sequence, four amino acid changes at positions 29, 62, 65 and 93 in the jacalin domain (which covers positions 1 to 151) were observed whereas a six amino acid deletion at the end of the C-terminal region is observed in the RTM1-4 sequence. All other accessions have the same RTM1 protein sequence than Col-0 (Fig. 1).

For RTM2, wide protein diversity was identified since 12 different protein sequences were observed, in addition to the one of Col-0. Among the 15 amino changes identified, two are in the HSP domain (from positions 16 to 118 according to the predicted secondary structure proposed in [12]), four in the α-helix region (from positions 119 to 223) and eight changes and a two amino acid deletion are in the C-terminal part of the protein. No variability is observed in the transmembrane domain located between positions 295 and 313 (Fig. 1). Up to three amino acid changes can be observed per protein sequence.

For RTM3, 11 protein sequences were identified in addition to the Col-0 one (Fig. 1). Among the 23 amino acid changes, 10 are located in the MATH domain (from amino acid 13 to 136) and 10 are located in the coiled coil (CC) domain (from amino acid 137 to 301, [14]). In addition a four amino acid insertion is present in the MATH domain in the St-0 and Pyl-1 accessions. Up to nine amino acid changes are observed per protein sequence. For the RTM3-12 sequence, found in accessions Blh-1 and Ge-1, a severely truncated protein is predicted, due to a stop codon identified at position 50.

The RTM protein pattern for each accession is presented in Table 1. Only two accessions (Jea and N13) share the same three RTM protein sequences than Col-0 and very few accessions have the same sequence pattern for all three proteins.

**Table 1:** RTM allelic pattern and infection phenotype with LMV isolates of each Arabidopsis accession.

Accessions	Accession origin	*RTM1* allele^a^	*RTM2* allele^a^	*RTM3* allele^a^	LMV-AF199^b^	LMV-AFVAR1^b^
Col-0	Poland	1	1	1	R	S
Jea	France	1	1	1	R	S
N13	Russia	1	1	1	R	S
Ws-2	Ukraine	1	9	1	R	S
Stw-0	Russia	1	3	1	R	S
Ita-0	Morocco	1	4	1	R	S
Kn-0	Lithuania	3	13	1	R	S
St-0	Sweden	1	1	2	R	R
Ge-0	Switzerland	1	1	8	R	R
Can-0	Canary Islands	1	1	9	R	R
Wu-0	Germany	1	9	3	R	R
Cvi-0	Cape Verde Islands	1	8	10	R	R
Mt-0	Libya	1	1	**4**	R	R
Ll-0	Spain	1	1	**4**	R	R
Gre-0	USA	1	9	**4**	R	R
Alc-0	Spain	1	11	**6**	R	R
Pyl-1	France	1	**6**	2	R	R
Nd-1	Germany	1	1	3	S	–
Ler-2	Poland	**4**	2	1	S	–
Edi-0	United Kingdom	1	1	**7**	S	–
Mh-1	Poland	1	1	**7**	S	–
Oy-0	Norway	1	1	**7**	S	–
Bur-0	Eire	1	1	**11**	S	–
Akita	Japan	1	7	**4**	S	–
Tsu-0	Japan	1	9	**6**	S	–
Ge-1	Switzerland	1	**10**	**12**	S	–
Sakata	Japan	1	**5**	**5**	S	–
Shahdara	Tadjikistan	1	**6**	**4**	S	–
Blh-1	Czech Republic	1	**6**	**12**	S	–
C24	Portugal	**2**	11	**4**	S	–
Bl-1	Italy	**2**	**5**	**4**	S	–
Ct-1	Italy	**2**	**12**	**4**	S	–

a Numbers in each column corresponding to each *RTM* allele refer to the *RTM* allele numbers described in Figure 1. The non-functional alleles are in bold.

b R: resistant to LMV systemic infection; S: susceptible to LMV systemic infection; - : not determined.

### Identification of Arabidopsis Accessions Permissive for Long Distance Movement of LMV

All 32 Arabidopsis accessions were inoculated with LMV-AF199, a LMV isolate previously shown to be restricted in Col-0 by the RTM resistance [8], [9]. For each accession at least two independent inoculation experiments were performed. LMV detection by ELISA, and by RT/PCR when the ELISA assay was negative, was performed 3 weeks after inoculation in un-inoculated inflorescence tissues. The results are shown in Table 1. Fifteen accessions supported systemic LMV-AF199 infection, indicating that the RTM resistance is not functional in them, whereas the remaining sixteen accessions showed resistance as no virus was detected in un-inoculated tissues. As previously observed [9], irrespective of the restriction or not of LMV-AF199 movement, no symptom was observed on any accessions. For two accessions, Gre-0 and St-0, a resistance phenotype was observed in this study contradicting previous analyses that showed a susceptibility phenotype to LMV-AF199 [9]. To try to explain these contradictory results, inoculations were performed in parallel for each accession using seeds coming from NASC (seed stock used by [9]) or from Versailles (this study). For Gre-0, the plants that developed from the NASC seeds (N1210) presented a different morphology than those from the Versailles stock and were found to be susceptible to LMV. For St-0, the seed stock from NASC (N1534) was apparently a mixture of two accessions. The plants with a morphology and development comparable to the Versailles ones were resistant to LMV while the plants with a different morphology were found susceptible. The Gre-0 and St-0 accessions from which *RTM* gene sequences were determined in the present work can therefore be safely considered resistant to LMV-AF199.

### Identification of Non Functional *RTM* Alleles in LMV Susceptible Accessions

To explain the LMV susceptibility phenotype of the fifteen accessions described in Table 1, we hypothesized that this phenotype is caused by the non-functionality of one or more RTM proteins in the resistance process in these accessions. To identify the corresponding *RTM* non-functional alleles, allelism tests were performed by crossing each of the 15 susceptible accessions with *rtm* mutant lines [10] carrying non-functional mutant alleles of either one of the three *RTM* genes. Given the dominant nature of the *RTM* resistance genes, the obtained F1 populations are expected to be fully resistant to LMV-AF199 if the tested accession and the tested mutant are affected in different *RTM* genes while they are expected to be susceptible if the two parents are affected in the same gene. The results of these experiments are presented in Tables 1 and S3.

In the case of RTM1, the *RTM1-2* and *RTM1-4* alleles carried respectively by C24 and La-er had previously been shown to be defective for restriction of TEV long distance movement [7], [10], [11]. These observations were confirmed with LMV for Ler-2 carrying RTM1-4 and Bl-1 and Ct-1 carrying RTM1-2, since the F1 plants produced from the crosses between Ler-2, Bl-1 or Ct-1 with the *rtm1-1* mutant line were all susceptible to LMV-AF199 (Tables 1 and S3).

In the case of RTM2, allelism tests were performed for the alleles corresponding to proteins RTM2-5, -6, -7, -10 and -12 found in susceptible accessions (Table 1). The *RTM2-2*, *RTM2-9* and *RTM2-11* alleles found in Ler-2, Tsu-0 and C24 respectively were not analysed since they had previously been shown to be functional for TEV long distance movement restriction [7], [10]. The results obtained showed that the *RTM2-5*, *-6*, -*10* and *-12* are not functional whereas *RTM2-7* is functional (Tables 1 and S3).

In the case of RTM3, allelism tests showed that the *RTM3-4*, *-5*, *-6*, *-7*, *-11* and *-12* alleles are not functional whereas *RTM3-3* is functional (Tables 1 and S3).

### The Functionality of the *RTM* Alleles is not Correlated to their Expression Level

In addition to their sequencing, we also analysed the expression of the three *RTM* genes in Col-0 and in 14 to 18 accessions (depending on the *RTM* gene) of the 31 accessions studied in this work, in order to assess if the functional versus non functional trait of some *RTM* alleles could be related to their expression level.

**Figure 2:**
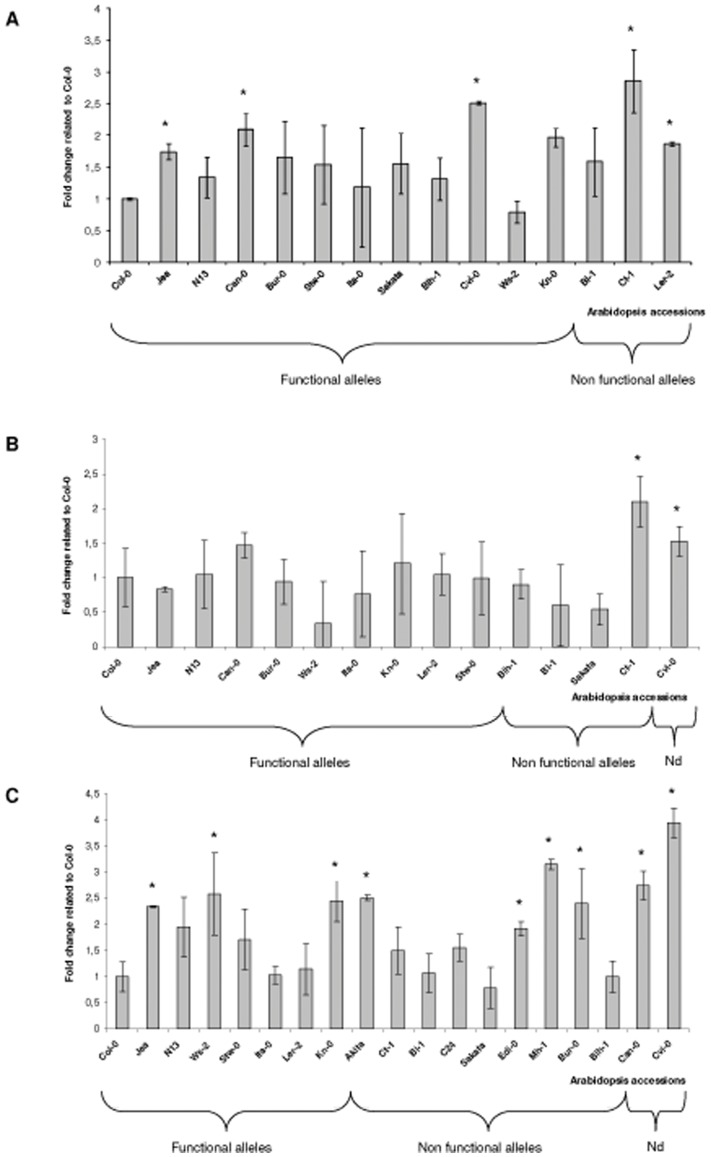
Q-RT-PCR analysis of the expression level of the three *RTM* genes in different Arabidopsis accessions. (**A**) *RTM1* expression; (**B**) *RTM2* expression; (**C**) *RTM3* expression. Fold change is determined relative to the value of Col-0 which is set arbitrarily at 1. The qPCR results are normalized to an ubiquitine-conjugating enzyme family gene (At2g36060). The graph represents the average values from three independent experiments involving 3 plants each. Bars represent SD of Ct values calculated using the Roche software. * : P<0.05; indicates that scoring values differ significantly from Col-0. Nd: not determined.

The analysis of the expression of the three *RTM* genes revealed significant differences between accessions but these differences could not be correlated to the functionality of the genes as non functional *RTM* alleles were in some cases more expressed than some functional ones and vice versa (Fig. 2). Even for the same *RTM* allele, variations could be observed between accessions. Significant differences in expression could even be observed between accessions sharing exactly the same RTM allelic composition as observed for Col-0, Jea and N13. It is worth noting than the *RTM* genes expression is not significantly modified during potyvirus infection (Schurdi-Levraud and Revers, unpublished).

### Evidence for the Involvement of New Genes in the RTM Resistance

The results presented above indicate that all LMV susceptible accessions have at least one non-functional *RTM* allele, with the exception of Nd-1 for which the three *RTM* genes appear to be functional (Tables 1 and S3). This observation suggests the existence of (an) additional factor(s) in Nd-1 compromising the resistance expected to be conferred by the presence of functional *RTM1*, *RTM2* and *RTM3* alleles.

In an attempt to identify this(ese) factor(s), a genetic analysis of susceptibility to LMV-AF199 was performed on a set of recombinant inbred lines produced between Col-5 (resistant) and Nd-1 [17] genotyped for a set of 93 markers [18]. Broad-sense heritability (H^2^) was 0.55. As shown in Table 2, two genetic loci located respectively on chromosome 1 (named *RTM4*) between markers nga280 and gen7463 and on chromosome 2 (named *RTM5*) between markers gen7259 and PhyB were identified as conferring susceptibility to LMV-AF199 systemic infection and do not correspond to the location of the *RTM1*, *RTM2* or *RTM3* genes. They respectively explained 15 and 24% of the phenotypic variation. No epistasis could be detected between these two loci.

**Table 2:** Genetic mapping of resistance loci using the Col-5xNd-1 RIL family.

Chromosome	Flanking-markers	Site (cM)^a^	Range (cM)	LOD	A^b^	SE^c^	*P*-value^c^	h^2^(a) (%)^d^
1	nga280-gen7463	73.8	70.2–79.8	5.00	−0.1624	0.4192	<0.0001	15.09
2	Gen7259-PhyB	29.2	23.8–34.9	3.29	−0.1922	0.4228	<0.0001	24.63

a distance between QTL and the first marker of the corresponding chromosome.

b additive effects, indicates the contribution of Nd alleles.

c the standard error of estimated QTL effect and P-value.

d heritability of additive effect, contribution explained by putative main-effect QTL.

### Identification of LMV-AF199 Resistant Accessions Susceptible to the RTM-breaking LMV-AFVAR1 Isolate

In order to evaluate whether the resistance observed in 16 of the 31 studied accessions is controlled by the RTM system or by other unknown mechanism(s), these accessions were challenged with LMV-AFVAR1, an LMV-AF199 point mutant able to overcome the RTM resistance in Col-0 and Ws-2 [15]. Seven accessions (N13, Jea, Stw-0, Kn-0, Ita-0, Col-0 and Ws-2) were found susceptible to LMV-AFVAR1, while all other tested accessions proved resistant to this LMV isolate (Table 1).

### Other members of the Small *RTM1*, *RTM2* and *RTM3* Gene Families are not Involved in the RTM Resistance

We noticed previously that the three *RTM* genes are co-expressed in several gene expression studies [14]. Using the Genevestigator database (https://www.genevestigator.com/gv/index.jsp; [19], [20], we identified other stimuli in response to which the three *RTM* genes are simultaneously up- (≥2.0 fold) or down-regulated (≤−2.0 fold). All three *RTM* genes are highly induced in suspension cells in presence of 1 µM brassinolide [21] and down-regulated in embryo endosperm from seeds maintained throughout on media containing either 20 mM abscisic acid (ABA) or 20 mM paclobutrazol (PAC, a gibberellin (GA) biosynthesis inhibitor) [22]. In addition, the three *RTM* genes are highly expressed in root phloem cells [23], which is not surprising as the *RTM* genes were previously shown to be specifically expressed in phloem tissues [13]. Using the Genevestigator Biomarker search tool we identified 56 genes sharing a similar expression pattern (Table S4). Among these genes, the three *RTM1* or *RTM2* homologous genes, At1g05770, At2g27140 and At3g10680 were identified. These three genes were also identified as *RTM* co-regulated genes using GeneMANIA (http://www.genemania.org, [24]), ATTED-II (http://atted.jp, [25]) or the expression angler tool from the Bio-Array Resource for Plant Biology (BAR, http://www.bar.utoronto.ca/ntools/cgi-bin/ntools_expression_angler.cgi, [26]) in the AtGenExpress seed and root sets (Table S4).

At1g05770 is the closest homolog to *RTM1* and At2g27140 and At3g10680 are the closest homologs to *RTM2*. The protein corresponding to At1g05770 presents 63% identity with RTM1 and these two genes are tandemly duplicated [27]. The proteins corresponding to At2g27140 (called Atuk in [12]) and At3g10680 present respectively 26% and 20% identity with RTM2. *RTM2* and At3g10680 are considered as duplicated genes [28], [29]. The co-regulation and common ancestry of these genes prompted experiments to evaluate the possibility that they could be involved in the RTM resistance. Although a similar comparison of expression profile could not be performed with the closest homologue of *RTM3*, At3g58360 (63% of amino acid identity with RTM3), as it is not represented on the microarrays used in the different studies, its potential contribution to the RTM resistance was also evaluated. After checking for homozygosity of the mutation and absence of gene expression (Fig. S2a,b), knock-out lines (all in a Col-0 background) N417974, N556006 and N606659, with T-DNA insertions at the At1g05770, At2g27140 and At3g58360 loci respectively (there is not Salk T-DNA insertion line for At3g10680), were challenged with LMV-AF199. All lines accumulated LMV in inoculated leaves but no viral accumulation was detected in inflorescence tissues (Fig. S2c), demonstrating that the RTM resistance was still active in these KO lines and, therefore, that these *RTM* genes-homologs are not involved in the RTM resistance at least in the Col-0 accession.

## Discussion

### Identification of RTM Protein Domains Involved in the Resistance Process

Screening of a panel of Arabidopsis accessions with LMV showed that some are permissive to the long distance movement of LMV, indicating that the RTM resistance is not active in these accessions. The analysis of the sequences of the three *RTM* genes from these accessions combined to allelism tests indicates that the LMV susceptibility trait of these accessions is related to the non-functionality of one or more *RTM* alleles. In addition we showed that this non-functionality is rather associated with amino acid changes in the RTM proteins than with changes in *RTM* gene expression. The positions of these amino acid changes thus allow the identification of mutations affecting the RTM resistance.

For RTM1, the 6 amino acid deletion at the end of the C-terminal end of RTM1-4 as well as the four amino acid changes in the jacalin domain of RTM1-2 demonstrate that both domains of the RTM1 protein are important for the resistance.

Regarding RTM2, four alleles (*RTM2-5*, *-6*, *-10* and *-12*) have been identified as non functional. RTM2-6 contains a unique amino acid change at position 176 in the α-helix [12], demonstrating the importance of this mutation and of this domain of the protein. RTM2-10 and RTM2-12 contain both three amino acid changes, including a shared pair of asparagines at positions 71 (HSP domain) and 188 (α helix) also present in the Ws-2 (RTM2-9) and C24 (RTM2-11) functional RTM2 proteins. Consequently, the non-functionality trait of RTM2-10 and RTM2-12 is most likely associated with the asparagine to lysine change at position 94 (HSP domain) for RTM2-10 and the serine to leucine change at position 287 (C-terminal domain) for RTM2-12. The involvement of the C-terminal domain of RTM2 is confirmed by the position of the two mutations (one amino acid change at position 225 and a two amino acid deletion at position 350) in RTM2-5 which are both in this domain. All together, these results suggest that the HSP domain, the α helix and the C-terminal end of RTM2 are all involved in the resistance mechanism.

Regarding RTM3, six non-functional alleles (*RTM3-4*, *-5*, *-6*, *-7*, -*11* and *-12*) have been identified. For the *RTM3-12* allele, this result was expected since it encodes a severely truncated protein limited to the first 49 amino acids of RTM3. Although it is not possible to evaluate their individual contribution, the positions of the amino acid changes in the RTM3-11 protein (position 12 in the N-terminal region and positions 20 and 77 in the MATH domain) suggest that either one of these domains is involved in the resistance process. The situation with the other non-functional alleles is more complex. However, an interesting situation is observed when comparing the *RTM3-3* (functional) and the *RTM3-4* (non-functional) alleles, since they only differ by a single amino acid change at position 127 in the MATH domain. RTM3-3 has a lysine at this position while RTM3-4 has a glutamate (as in the Col-0 functional form). Taken together these observations indicate that a mutation at position 127 is able to compensate the detrimental effect of one or more of the 7 mutations separating the RTM3-4 and Col-0 forms. Combined with the previous observation that the *rtm3-1* EMS mutant contains a single change in the CC domain [14], we can then conclude that the RTM3 MATH and CC domains are both involved in the resistance mechanism.

All together, these results indicate that mutations in most of the RTM protein domains lead to the non-functionality of these proteins for the resistance to LMV. Most of the RTM protein domains are known to be involved in protein-protein interactions, such as the jacalin domain of RTM1 involved in the tetrameric structure of jacalin [30], the HSP domain of RTM2 involved in the heterooligomeric structure of small HSPs [31] and the MATH domain of RTM3 involved in the trimeric structure of TRAF proteins [32]. The coiled-coil domain in the C-terminal part of RTM3 [14] and the α-helix of RTM2 [12], which is also predicted to form a coiled-coil domain (Fig. S3), could also be involved in protein-protein interaction. In addition, we showed self-interaction for RTM1 and RTM3 as well as interaction between RTM1 and RTM3 [14]. Then it might be suggested that the mutations in the RTM non-functional proteins disrupt interactions necessary for the functionality of these proteins.

Another suggestion would be that these mutations alter the stability of the RTM proteins either by destabilizing their structure or by increasing their degradation. More investigation will be necessary to test these hypotheses as well as to determine the putative role of each of the RTM protein domains in the resistance process.

### New Loci are Involved in the RTM Resistance

Two new *RTM* loci (*RTM4* and *RTM5*) have been identified using a genetic mapping approach in a RIL population produced between Col-5 and Nd-1, though these genes were not identified in the genetic screen of chemically induced Col-0 mutants carried out with TEV [10]. The involvement of these loci increases the level of complexity of the RTM resistance and their cloning will be an important step to better understand this resistance mechanism.

The genetic analysis of other crosses between Col-0 and other LMV susceptible accessions could also be useful to determine if yet other genes are involved in the RTM resistance.

### Preponderance of the RTM Resistance in *A. thaliana*

Inoculation of all the accessions shown to be resistant to LMV-AF199 with a RTM-breaking LMV isolate (LMV-AFVAR1) indicate that seven of them (41%) are susceptible to this isolate, strongly suggesting that their resistance to LMV-AF199 is controlled by the RTM genes (Table 1). That was expected for Jea and N13 which have the Col-0 RTM allelic pattern. Regarding the other accessions, these data indicate that the *RTM1-3* allele present in Kn-0 and the *RTM2-3*, *-4*, *-9* and *-13* alleles present in Stw-0, Ita-0, Ws-2 and Kn-0 respectively are functional alleles. Of course, we cannot exclude that LMV-AFVAR1 is able to overcome a RTM-independent resistance but that would be very surprising as this isolate differs from LMV-AF199 by a single amino acid change in the N-terminal domain of its coat protein. Regarding the LMV-AF199 resistant accessions which are also resistant to the RTM-breaking LMV-AFVAR1, two hypotheses can be proposed: the involvement of other resistance mechanism(s) or an ability of the *RTM* alleles they harbour to control this variant. The first hypothesis appears the most likely for 5 accessions (Pyl-1, Gre-0, Mt-0, Ll-0 and Alc-0) that have at least one *RTM* allele shown to be non-functional (Table 1). The genetic characterization of these new resistances would be of a great interest for the study of the Arabidopsis/LMV interactions.

### Is the RTM Resistance Controlled by Hormones?

The expression of all three *RTM* genes is strongly modified by several hormonal stimuli, independently of viral infection. In particular brassinosteroids and GA lead to *RTM* genes up-regulation while ABA treatment leads to their down-regulation. The function(s) of the *RTM* genes that might be controlled by these different hormones need(s) to be investigated. The description of an *RTM* co-regulated gene network allowed the identification of a panel of genes which might be associated with biological processes involving the *RTM* genes. However, our results rule out the involvement in the RTM resistance of the co-regulated *RTM* genes homologs, suggesting that their co-regulation might be associated with another cellular process. The observation that the *RTM* genes are strongly regulated in response to various hormonal stimuli might provide an avenue to the understanding of their biological function and indicate that the phenotyping of the LMV-Arabidopsis interaction under modified hormonal status might be worth pursuing.

### Is the RTM Resistance a Novel form of Plant Antiviral Defense Response?

It has been suggested that the RTM genes can be considered as an atypical class of disease resistance R genes [33]. Indeed, their study reveals intriguing and striking similarities with the dominant NBS-LRR R genes. First, as the classical R proteins, many RTM protein domains are involved in protein-protein interaction and some of them are known to be involved in plant defense or chaperone activity as the jacalin domain present in RTM1 [34], [35] or the hsp domain identified in RTM2 [36]. Second, the cluster organisation of *RTM3* and the *RTM3*-like genes in the *Arabidopsis* genome showing evidence of gene duplication and deletion events presents similarity to the cluster organisation of the R genes. Third, the potyvirus CP could be considered as the avirulence factor the recognition of which might involve a RTM multi-protein complex. Fourth, the RTM-mediated resistance might be controlled by hormones as suggested by our study as the R-mediated resistance [37]. However, in the RTM-mediated resistance, there is no HR, production of SA, or induction of SAR. In addition, the RTM-mediated resistance is not race specific as is the case for most of the R-mediated resistances since the same RTM genes control the systemic infection of several potyviruses [7], [8]. The RTM genes may simply act as inhibitory factors of the potyvirus long distance movement as the *Tm-1* resistance gene from tomato which encodes a factor which interact with *Tomato mosaic virus* (ToMV; genus *Tobamovirus*) replication proteins causing inhibition of the ToMV replication without inducing a hypersensitive reaction [38], [39].

Thus, the RTM resistance may be considered as a novel form of plant defence response acting in phloem-associated tissues against viruses.

Although the results presented here increase our knowledge on this original resistance, there is still a long way to precisely understand the mechanism(s) underlying the RTM resistance. The characterization of the role of each *RTM* gene and their protein domains in the resistance process, the identification of the *RTM4* and *RTM5* genes and the assessment of the putative influence of plant hormones are the new challenges for the coming years.

## Materials and Methods

### Plant Material

Accessions included in the Versailles core 24 collection [16], a collection which covers 96% of the genetic diversity of a worldwide sample of 95 Arabidopsis accessions, were obtained from the INRA Versailles (http://dbsgap.versailles.inra.fr/vnat/). Other accessions and the Col-5×Nd-1 Recombinant Inbred Lines (RILs, [17]) were obtained from the Nottingham Arabidopsis stock Centre (NASC, http://nasc.nott.ac.uk/). The Versailles and NASC references of the accessions are indicated in Table S1.

The F1 populations produced between the rtm mutant lines and the Arabidopsis accessions were controlled prior to the inoculation experiments using the microsatellite marker MSAT2.5 described in [40], which is polymorphic between Col-0 and each of the core-collection accessions, and CAPS or dCAPS markers developed in this study to control the identity of the mutations in the *rtm* mutant genes (Table S5).

Arabidopsis T-DNA insertion lines in the Col-0 background were obtained from NASC (line N613698 for At3g58360, line N417974 for At1g05770; line N556006 for At2g27140). For lines coming from the Salk Institute (N613698 and N556006), the T-DNA insertion sites were confirmed by PCR using primers designed from the SIGnAL T-DNA Verification Primer Design program (http://signal.salk.edu/tdnaprimers.2.html) and the T-DNA left border-specific primers LBa1 5′-TGGTTCACGTAGTGGGCCATCG-3′ or LBb1 5′ GCGTGGACCGCTTGCTGCAACT-3′. For the At1g05770 T-DNA line (N417974) coming from Gabi-Kat, the T-DNA specific primer 5′-ATATTGACCATCATACTCATTGC-3′ (GK-TDNA) was used in association with the At1g05770-1 and At1g05770-2 primers (Table S6). Genomic DNA used for PCR for each line was extracted from Arabidopsis young leaves using the NucleoSpin® Plant kit (Macherey-Nagel, Düren, Germany).

Absence of expression of the targeted genes in the appropriate T-DNA lines was checked as described in [14], using the gene specific oligonucleotides At2g27140-1 and At2g27140-2 for At2g27140, At1g5770-1 and At1g5770-2 for At1g05770 and At3g58360-1 and At3g58360-2 for At3g58360 (Table S6). Complementary DNAs from total RNAs of wild-type Col plants was used as positive control. The *RTM1* gene specific oligonucleotides RTM1-int5 and RTM1-3 (Table S6) were also used to amplify the RTM1 cDNA as a positive control for the cDNA synthesis from the KO lines. Genomic DNA was used as control to show that total RNA extracts were DNA-free.

### Virus Inoculation and Detection

Inoculation of the Arabidopsis plants with LMV-AF199 [41] or its RTM-breaking variant LMV-AFVAR1 [15] were performed as described in [9]. Enzyme-linked immunosorbent assay (ELISA) and RT-PCR used to detect LMV in inoculated leaves and inflorescence tissues were performed as described in [9], [42].

### Gene Sequencing

The sequenced regions are from the start codon to the stop codon and are respectively 644 nucleotides (nt), 1174 nt and 1219 nt long for *RTM1*, *RTM2*, *RTM3*. One µl of a ten-fold dilution of genomic DNA was used for PCR amplifications performed in 50-µl reactions containing 0.5 units of DyNazyme™ EXT DNA Polymerase (Finnzymes, Espoo, Finland) and 1 µM of primers. All the pairs of primers used for PCR amplification of each *RTM* gene are described in Table S6. All primers were chosen in order not to amplify the *RTM*-homologuous genes. The cycling conditions were 35 cycles at 92°C 30 s, 52°C 30 s, 72°C 2 min after an initial denaturation at 95°C for 3 min using an iCycler thermal cycler (Bio-Rad Laboratories, Hercules, CA, USA). Automated DNA sequencing of PCR products (from two independent PCR products) was performed at GENOME Express (Meylan, France).

Genbank accession numbers for all RTM sequences produced in this study are provided in Table S7.

### Sequence Analysis

The sequences were aligned using ClustalW [43], which generates and uses a distance dendrogram [44] to construct multiple sequence alignments.

Sequence polymorphisms in *A. thaliana* were analyzed using the DnaSP program version 5.10.0 [45]. Nucleotide variation was estimated as nucleotide diversity (π, [46]) and 4 *N*m (θ, [47]). Standard errors for nucleotide diversity were obtained by the bootstrap method implemented in the MEGA software version 4.0 [48].

### Gene Expression Analysis

Rosette leaves from 4-week-old plants were harvested, quickly frozen in liquid nitrogen and ground using a Retsch MM301 grinder. RNA was isolated by using the SV Total RNA isolation kit (Promega) according to the manufacturer’s instructions. RNA samples were treated with TURBO™ DNase (Ambion) to remove contaminating genomic DNA according to the manufacturer’s instructions. PCR amplification of *RTM1* using RTM1 specific primers (Table S6) was then performed to check that the samples were DNA-free. Reverse transcription was done by using 1 µg of total RNA and Superscript III Reverse Transcriptase (Invitrogen). Q-RT-PCR was performed on a Light Cycler 480 II machine (Roche Diagnostics) using Absolute Blue QPCR SYBR Green reagents (Thermo Scientific). The primers used for Q-RT-PCR analysis were RTM1F and RTM1R for RTM1, RTM2F and RTM2R for RTM2, RTM3F and RTM3R for RTM3, At2g36060F and At2g36060R for At2g36060 (ubiquitin E2 variant 1c protein) used as an internal control (Table S6). PCR was performed using the following cycling conditions: 95°C for 15 min, and 40 cycles of 95°C for 30 s, 59°C for 30 s and 72°C for 30 s. Three independent Q-RT-PCR experiments were performed, testing three plants per accession in each experiment.

Relative expression was calculated using the Efficiency method (Roche) in comparison with the endogenous control. Fold change was determined relative to the value of Col-0, which was set at 1. Kruskal-Wallis test (P<0.05) was performed to assess significant differences in *RTM* gene expression between accessions and Columbia used as a reference.

### Genetic Mapping

A set of 96 RILs (5 plants per line) derived from the crosses between Col-5 and Nd-1 (Holub & Beynon 1997) were phenotyped 21 days after inoculation with LMV-AF199. Systemic leaves and stems were sampled. The virus was detected by ELISA as described above. Optical density values were used as data. Values under 3 times the blank value were considered negative whereas values above this threshold were considered as positive. Phenotypic values were then collected as 0 when no virus could be detected and 1 when plants were considered as positive. Linkage mapping was performed using MAPMAKER/Exp version 3.0 b [49]. QTLs were mapped by using QTLNetwork 2.1 [50] based on a mixed-model composite interval mapping method (MCIM). Genome scan was performed using a 10 cM testing window, a 0.1 cM walk speed and a 0.5 cM filtration window. To control the experimental type I error, a critical *F* value was calculated using 1000 permutations test. QTL effects and QTL confidence intervals were estimated with a Bayesian method (Gibbs sample size = 20,000). Composite interval mapping (CIM) using Windows QTL Cartographer, version 2.5 [51] was used to determine LOD score values for each QTL. Standard model was used to scan the genome at 2-cM intervals and using a window size of 10 cM. Five markers were selected as cofactors, using the forward-backward regression method. One thousand permutations were used to determine LOD significance levels (p = 0.01).

## Supporting Information

Figure S1 **Polymorphic sites in the** ***RTM*** **genes genomic sequences.**
(DOC)

Figure S2 **Genotyping and LMV infection phenotyping of the** ***RTM*** **homologous genes KO lines.**
(DOC)

Figure S3 **Coiled coil structure prediction in the RTM2 long α helix.**
(DOC)

Table S1 **Name and accession number of Arabidopsis accessions used in the present work.**
(DOC)

Table S2 **Patterns of nucleotide variation in the** ***RTM*** **genes.**
(DOC)

Table S3 **Allelism test by crossing susceptible accessions to LMV-AF199 with the** ***rtm*** **mutants.**
(DOC)

Table S4 **List of the** ***RTM*** **co-regulated genes.**
(DOC)

Table S5 **Markers to control mutation in the** ***RTM*** **mutant genes.**
(DOC)

Table S6 **List of the primers used in this study.**
(DOC)

Table S7 **Genbank accession number for each RTM sequence produced in this study.**
(DOC)
