# Supplementary material for: The RTM Resistance to Potyviruses in Arabidopsis thaliana: Natural Variation of the RTM Genes and Evidence for the Implication of Additional Genes
Source: PLoS One. 2012 Jun 18;7(6):e39169. doi: 10.1371/journal.pone.0039169 (PMC3377653; doi:10.1371/journal.pone.0039169)
Supplement: Figure S1 — Polymorphic sites in the RTM genes genomic sequences. (DOC) [file pone.0039169.s001.doc]

**Figure S1 : polymorphic sites in the *RTM* genes genomic sequences.**

The number above each polymorphic site corresponds to their position in the genomic sequence of each *RTM* genes in Col-0. The red parts correspond to introns. “.” indicate an identity with the Col-0 one sequence. “-“ indicates a deletion (gap) as compared to the Col-0 sequence.

(a) polymorphic sites in the *RTM1* gene. (b) polymorphic sites in the *RTM2* gene. (c) polymorphic sites in the *RTM3* gene. In *RTM3,* Insert 1 is between positions 138 and 139 of the Col-0 sequence, insert 2 between positions 221 and 222, insert 3 between positions 247 and 248, insert 4 between positions 395 and 396 and insert 5 between positions 408 and 409.
