# Supplementary material for: The RTM Resistance to Potyviruses in Arabidopsis thaliana: Natural Variation of the RTM Genes and Evidence for the Implication of Additional Genes
Source: PLoS One. 2012 Jun 18;7(6):e39169. doi: 10.1371/journal.pone.0039169 (PMC3377653; doi:10.1371/journal.pone.0039169)
Supplement: Figure S2 — Genotyping and LMV infection phenotyping of the RTM homologous genes KO lines. (DOC) [file pone.0039169.s002.doc]

**Figure S2**. **Genotyping and LMV infection phenotyping of the *RTM* homologous genes KO lines.**

KO-1: At1g05770 KO line (N417974); KO-2: At2g27140 KO line (N556006); KO-3: At3g58360 KO line (N613698) ; L: 1kb Ladder; H2O: negative control without genomic DNA.

(a). Genotyping of three KO lines by PCR on genomic DNA. For each KO line, a PCR performed with the gene specific primers and another PCR performed with a T-DNA specific primer and one of the gene specific primer are shown. For each KO line, the number indicates the number of the analysed KO plant (see Materials and Methods).

(b). RT-PCR amplification of *RTM1* (as control) and RTM homologous transcripts from total RNA from wild-type (Col-0) and KO lines. gDNA: genomic DNA; cDNA: complementary DNAL.

(c). RT-PCR of a 277-bp fragment of LMV RNA from LMV-inoculated KO-1, KO-2 and KO-3. LMV-inoculated *RTM3* KO and *rtm3* mutants (Cosso*n et a*l. 2010) are used as susceptible lines. Mock corresponds to mock-inoculated Col-0 plants which are used as negative control.
