# Supplementary material for: The RTM Resistance to Potyviruses in Arabidopsis thaliana: Natural Variation of the RTM Genes and Evidence for the Implication of Additional Genes
Source: PLoS One. 2012 Jun 18;7(6):e39169. doi: 10.1371/journal.pone.0039169 (PMC3377653; doi:10.1371/journal.pone.0039169)
Supplement: Figure S3 — Coiled coil structure prediction in the RTM2 long α helix. (DOC) [file pone.0039169.s003.doc]

(a)

(119)EAAALEKAAKLEEKRLLEESRRKEKEEEEAKQMKKQLLEEKEALIRKLQEEAKAKEEAEMRKLQEEAKAKEEAAAKKLQEEIEAKEKLEERKLEERRLEERKLEDMKLAEEAKLKKIQERKSVDESGEKEKILKPE(254)

(b)


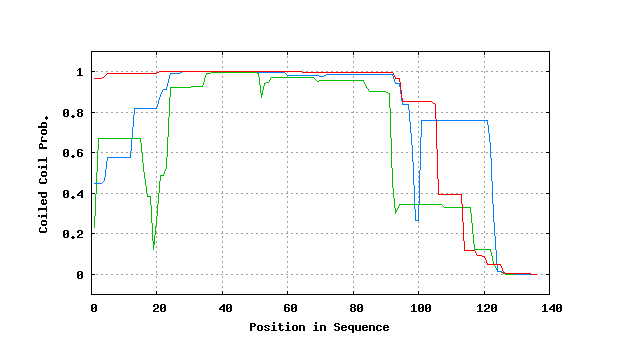


(c)

1E[42.5]g 2A[55.4]a 3A[65.2]b 4A[73.3]c 5L[80.5]d 6E[83.8]e 7K[88.5]f **8A[90.7]g 9A[92.9]a 10K[94.2]b 11L[94.9]c 12E[96.7]d 13E[97.4]e 14K[98.5]f 15R[99.1]g 16L[99.5]a 17L[99.6]b 18E[99.7]c 19E[99.8]d 20S[99.8]e 21R[99.8]f 22R[99.9]g 23K[99.9]a 24E[100.]b 25K[100.]c 26E[100.]d 27E[100.]e 28E[100.]f 29E[100.]g 30A[100.]a 31K[100.]b 32Q[100.]c 33M[100.]d 34K[100.]e 35K[100.]f 36Q[100.]g 37L[100.]a 38L[99.9]b 39E[99.9]c 40E[99.9]d 41K[99.8]e 42E[99.8]f 43A[99.6]g 44L[99.5]a 45I[99.2]b 46R[99.2]c 47K[99.2]d 48L[99.1]e 49Q[99.1]f 50E[99.3]g 51E[99.0]a 52A[98.8]b 53K[99.0]c 54A[99.1]d 55K[99.2]e 56E[99.3]f 57E[99.6]g 58A[99.5]a 59E[99.6]b 60M[99.3]c 61R[99.3]d 62K[99.4]e 63L[99.4]f 64Q[99.6]g 65E[99.7]a 66E[99.8]b 67A[99.7]c 68K[99.7]d 69A[99.6]e 70K[99.6]f 71E[99.6]g 72E[99.3]a 73A[99.3]b 74A[99.4]c 75A[99.5]d 76K[99.7]e 77K[99.7]f 78L[99.7]g 79Q[99.8]a 80E[99.9]b 81E[99.9]c 82I[99.9]d 83E[99.9]e 84A[100.]f 85K[100.]g 86E[100.]a 87K[100.]b 88L[100.]c 89E[100.]d 90E[100.]e 91R[100.]f 92K[100.]g 93L[100.]a 94E[100.]b 95E[100.]c 96R[100.]d 97R[100.]e 98L[99.9]f 99E[99.9]g 100E[99.9]a 101R[99.9]b 102K[99.9]c 103L[99.8]d 104E[99.8]e 105D[99.7]f 106M[99.7]g 107K[99.6]a 108L[99.5]b 109A[99.4]c 110E[99.4]d 111E[99.3]e 112A[99.2]f 113K[98.9]g 114L[98.5]a 115K[98.2]b 116K[97.5]c 117I[96.4]d 118Q[95.3]e 119E[92.8]f** 120R[86.3]g 121K[75.9]a 122S[64.3]b 123V[52.9]c 124D[46.8]d 125E[44.2]e 126S[35.1]f 127G[27.1]e 128E[25.3]f 129K[23.8]g 130E[20.6]a 131K[15.9]b 132I[12.1]c 133L[10.5]d 134K[07.7]e 135P[02.3]f 136E[01.8]g

**Figure S3:** **Coiled coil structure prediction in the RTM2 long α helix**. (a) Amino acid sequence of the long α helix (from position 119 to 254 in the RTM2 protein). (b) Output of the the PCOIL prediction program (Gruber *et al.* 2005; Lupas 1996; http://toolkit.tuebingen.mpg.de/pcoils). In green: window of 14 amino acids, in blue: window of 21 amino acids, in red: window of 28 amino acids. (c) With the Marcoil program (Delorenzi and Speed 2002; http://www.isrec.isb-sib.ch/webmarcoil/webmarcoilC1.html). The table indicates for each amino acid along the RTM2 sequence the coiled-coil probability in percent (in bracket) and the heptad phase (a to g). In bold, the region of RTM2 with the highest coiled-coil probability.

References

Delorenzi M., Speed T (2002) An HMM model for coiled-coil domains and a comparison with PSSM-based predictions. *Bioinformatics* 18(4), 617-625

Gruber M, Söding J. & Lupas A.N. (2005) REPPER—repeats and their periodicities in fibrous proteins. *Nucleic Acids Research* 33, W239-W243

Lupas A.N. (1996) Prediction and analysis of coiled-coil structures. *Methods in Enzymology* **266,** 513–525
