# Supplementary material for: The RTM Resistance to Potyviruses in Arabidopsis thaliana: Natural Variation of the RTM Genes and Evidence for the Implication of Additional Genes
Source: PLoS One. 2012 Jun 18;7(6):e39169. doi: 10.1371/journal.pone.0039169 (PMC3377653; doi:10.1371/journal.pone.0039169)
Supplement: Table S1 — Name and accession number of Arabidopsis accessions used in the present work. (DOC) [file pone.0039169.s004.doc]

**Table S1: Name and accession number of Arabidopsis accessions used in the present work**

| **Accessions** | **Versailles (AV) or NASC number (N)** |
| --- | --- |
| Col-0 | N1092 |
| Nd-1 | N1636 |
| Ll-0 | N1338 |
| Wu-0 | N1614 |
| Ws-2 | N1601 |
| Ge-1 | N1188 |
| C24 | N906 |
| Ler-2 | N8581 |
| Pyl-1 | 8AV |
| Jea | 25AV |
| Bl-1 | 42AV |
| St-0 | 62AV |
| Kn-0 | 70AV |
| Edi-0 | 83AV |
| Tsu-0 | 91AV |
| Stw-0 | 92AV |
| Mt-0 | 94AV |
| Ge-0 | 101AV |
| Ita-0 | 157AV |
| Ct-1 | 162AV |
| Can-0 | 163AV |
| Cvi-0 | 166AV |
| Bur-0 | 172AV |
| Alc-0 | 178AV |
| Blh-1 | 180AV |
| Gre-0 | 200AV |
| Mh-1 | 215AV |
| Oy-0 | 224AV |
| Shahdara | 236AV |
| Akita | 252AV |
| Sakata | 257AV |
| N13 | 266AV |
