# Supplementary material for: The RTM Resistance to Potyviruses in Arabidopsis thaliana: Natural Variation of the RTM Genes and Evidence for the Implication of Additional Genes
Source: PLoS One. 2012 Jun 18;7(6):e39169. doi: 10.1371/journal.pone.0039169 (PMC3377653; doi:10.1371/journal.pone.0039169)
Supplement: Table S2 — Patterns of nucleotide variation in the RTM genes. (DOC) [file pone.0039169.s005.doc]

**Table S2. Patterns of nucleotide variation in the *RTM* genes.**

Sites with alignment gaps were excluded from the analysis.
