# Supplementary material for: The RTM Resistance to Potyviruses in Arabidopsis thaliana: Natural Variation of the RTM Genes and Evidence for the Implication of Additional Genes
Source: PLoS One. 2012 Jun 18;7(6):e39169. doi: 10.1371/journal.pone.0039169 (PMC3377653; doi:10.1371/journal.pone.0039169)
Supplement: Table S3 — Allelism test by crossing susceptible accessions to LMV-AF199 with the rtm mutants. (DOC) [file pone.0039169.s006.doc]

**Table S3. Allelism test by crossing susceptible accessions to LMV-AF199 with the *rtm* mutants**

| **F1^a^** | ***RTM* allele number^b^** | **F1 phenotype**  **after inoculation with LMV-AF199^c^** |
| --- | --- | --- |
| *rtm1* x Bl-1/ Ct-1 | *RTM1-2* | S |
| *rtm1* x Ler-2 | *RTM1-4* | S |
|  |  |  |
| *rtm2* x Sakata/Bl-1 | *RTM2-5* | S |
| *rtm2* x Shahdara/Blh-1 | *RTM2-6* | S |
| *rtm2* x Akita | *RTM2-7* | R |
| *rtm2* x Ge-1 | *RTM2-10* | S |
| *rtm2* x Ct-1 | *RTM2-12* | S |
|  |  |  |
| *rtm3* x Nd-1 | *RTM3-3* | R |
| *rtm3* x Shahdara/Bl-1/Ct-1 | *RTM3-4* | S |
| *rtm3* x Sakata | *RTM3-5* | S |
| *rtm3* x Tsu-0 | *RTM3-6* | S |
| *rtm3* x Oy-0/ Edi-0/ Mh-1 | *RTM3-7* | S |
| *rtm3* x Bur-0 | *RTM3-11* | S |
| *rtm3* x Blh-1 | *RTM3-12* | S |
|  |  |  |
|  |  |  |
| Negative controls |  |  |
|  |  |  |
| *rtm1* x Col-0 | *RTM1-1* | R |
| *rtm2* x Col-0 | *RTM2-1* | R |
| *rtm3* x Col-0 | *RTM3-1* | R |
| *rtm1* x Akita | *RTM1-1* | R |
| *rtm2 x Oy-0* | *RTM2-1* | R |
| *rtm1 x rtm2* | *RTM1-1 / RTM2-1* | R |
| *rtm1 x rtm3* | *RTM1-1 / RTM3-1* | R |
| *rtm2 x rtm3* | *RTM2-1 / RTM3-1* | R |
| *rtm1* x Ws-2 | *RTM1-1* | R |
| *rtm2* x Ws-2 | *RTM2-9* | R |
| *rtm3* x Ws-2 | *RTM3-1* | R |

^a^ Crosses between the different rtm mutant lines and the LMV-susceptible accessions

^b^ *RTM* alleles described in Table 1 present in the corresponding susceptible accessions

^c^ R: resistant to LMV systemic infection; S: susceptible to LMV
