# Supplementary material for: The RTM Resistance to Potyviruses in Arabidopsis thaliana: Natural Variation of the RTM Genes and Evidence for the Implication of Additional Genes
Source: PLoS One. 2012 Jun 18;7(6):e39169. doi: 10.1371/journal.pone.0039169 (PMC3377653; doi:10.1371/journal.pone.0039169)
Supplement: Table S4 — List of the RTM co-regulated genes. (DOC) [file pone.0039169.s007.doc]

**Table S4: List of the *RTM* co-regulated genes**

^a^ gene list of *RTM* co-regulated genes using the Genevestigator Biomarker search tool (https://www.genevestigator.com/gv/index.jsp ); ^b^ *RTM* co-regulated genes from http://www.genemania.org; ^c^ *RTM* co-regulated genes from [http://atted.jp](http://atted.jp/); ^d^ *RTM* co-regulated genes from AtGenExpress (http://www.bar.utoronto.ca/ntools/cgi-bin/ntools_expression_angler.cgi). “x” indicates that the corresponding gene is found with the corresponding tool.
