# Supplementary material for: The RTM Resistance to Potyviruses in Arabidopsis thaliana: Natural Variation of the RTM Genes and Evidence for the Implication of Additional Genes
Source: PLoS One. 2012 Jun 18;7(6):e39169. doi: 10.1371/journal.pone.0039169 (PMC3377653; doi:10.1371/journal.pone.0039169)
Supplement: Table S5 — Markers to control mutation in the RTM mutant genes. (DOC) [file pone.0039169.s008.doc]

**Table S5: Markers to control mutation in the *RTM*** mutant genes

| **Marker** | **Sequence of the used primer pairs** | **Marker type^a^** | **Restriction enzyme** | **Size Wt (pb)** | **Size mutant (pb)** |
| --- | --- | --- | --- | --- | --- |
| CAPS rtm1 | RTM1-3  RTM1-int5 | CAPS | PsiI | 340 | 143 / 197 |
| dCAPS rtm2 | 5’ACGAGGACTTTGTCCCCAAATCCGAATGGAAATAT  5’TTATCATCTTCGAGCTGTGC | dCAPS | SspI | 199 | 33 / 165 |
| dCAPS rtm3 | 5’GATAGGAGCTTTTGCAGTCGACACTTTTGCCTTCC  5’AGAAATGGAGGAAGAGTTGAA | dCAPS | MnlI | 103 | 44 / 58 |

a CAPS: Cleaved Amplified Polymorphic Sequence; dCAPS: derived Cleaved Amplified Polymorphic Sequence. dCAPS were designed according to Neff et al. (2002).

Reference:

Neff MM, Turk E and Kalishman M (2002) Web-based Primer Design for Single Nucleotide Polymorphism Analysis. Trends in Genetics, 18 613-615.
