# Supplementary material for: The RTM Resistance to Potyviruses in Arabidopsis thaliana: Natural Variation of the RTM Genes and Evidence for the Implication of Additional Genes
Source: PLoS One. 2012 Jun 18;7(6):e39169. doi: 10.1371/journal.pone.0039169 (PMC3377653; doi:10.1371/journal.pone.0039169)
Supplement: Table S6 — List of the primers used in this study. (DOC) [file pone.0039169.s009.doc]

**Table S6: List of the primers used in this study**

| **Primer name** | **Primer sequence** |
| --- | --- |
| At1g5770-1 | 5’-TCTCATCATGTAACAAGATTATCC-3’ |
| At1g5770-2 | 5’-TGATATAAACGCATGTAGAGGCTG-3’ |
| At2g27140-1 | 5’-CAAGTAACCACCACGAGGAAG-3’ |
| At2g27140-2 | 5’-GGAACATGTAAGGTCTTATTTTACTTC-3’ |
| At3g58360-1 | 5’ ATGGGGAAGCAACTTGCTAA-3’ |
| At3g58360-2 | 5’-GCAAACGAACAGACCAAGG-3’ |
| RTM1-5 | 5’-GGATAGGCATATTCCAATCTGAGAC-3’ |
| RTM1-3 | 5’-ATCAGCAGAATAGAGTCGTATACAA-3’ |
| RTM1-int3 | 5’-TGTAGTCCCGAGGCATCATAAG-3’ |
| RTM1-int5 | 5’-AACTATCCGCATGAGTACATAAC-3’ |
| RTM2-5 | 5’-TTTAACCAGTTCATAAGAAAGAGGA-3’ |
| RTM2-3 | 5’-TACACATTCTTCAATTTGCACAACA-3’ |
| RTM2-int5 | 5’-GAGGAAAGGAGACTTGAGGAG-3’ |
| RTM2-int5.1 | 5’-CTGGAGAACGTCCCTTGGCTAATC-3’ |
| RTM2-int3 | 5’-GTTCTAGAAGTTTCTGGAAGATAAG-3’ |
| RTM3-3 | 5’-AGCGTATTCGATATGCAAGTC-3’ |
| RTM3-5 | 5’-CGATTCGTATCCCTCATGTAC-3’ |
| RTM3-int5 | 5’-ACACCACAAAAGCACCATATC-3’ |
| RTM3-int3 | 5’-TTCAACAATCACGGAAACTG-3’ |
| RTM1F | 5′-actgggacgaaggatctcac-3’ |
| RTM1R | 5′-tccatcacgaactggaattg-3’ |
| RTM2F | 5′-cgaggtgttcactgttccac-3’ |
| RTM2R | 5′-ttaggcatggtgatggtgag-3’ |
| RTM3F | 5′-ctgagatttgggttctttgga-3’ |
| RTM3R | 5′-caacaatcacggaaactgtgga-3’ |
| At2g36060F | 5′-cctacctttgaggggcttct-3’ |
| At2g36060R | 5′-gaagtcgtgagacagcgttg-3’ |
