# Supplementary material for: The RTM Resistance to Potyviruses in Arabidopsis thaliana: Natural Variation of the RTM Genes and Evidence for the Implication of Additional Genes
Source: PLoS One. 2012 Jun 18;7(6):e39169. doi: 10.1371/journal.pone.0039169 (PMC3377653; doi:10.1371/journal.pone.0039169)
Supplement: Table S7 — Genbank accession number for each RTM sequence produced in this study. (DOC) [file pone.0039169.s010.doc]

**Table S7: Genbank accession number for each RTM sequence produced in this study**

| Arabidopsis accessions | Genbank accession Number for *RTM1* | Genbank accession Number for *RTM2* | Genbank accession Number for *RTM3* |
| --- | --- | --- | --- |
| Pyl-1 | FR681968 | FR682045 | FR681937 |
| Jea | FR681969 | FR682046 | FR681938 |
| Bl-1 | FR681970 | FR682047 | FR681939 |
| St-0 | FR681971 | FR682048 | FR681940 |
| Kn-0 | FR681972 | FR682049 | FR681941 |
| Edi-0 | FR681973 | FR682050 | FR681942 |
| Tsu-0 | FR681974 | FR682051 | FR681943 |
| Stw-0 | FR681975 | FR682052 | FR681944 |
| Mt-0 | FR681976 | FR682053 | FR681945 |
| Ge-0 | FR681977 | FR682054 | FR681946 |
| Ita-0 | FR681978 | FR682055 | FR681947 |
| Ct-1 | FR681979 | FR682056 | FR681948 |
| Can-0 | FR681980 | FR682057 | FR681949 |
| Cvi-0 | FR681981 | FR682058 | FR681950 |
| Bur-0 | FR681982 | FR682059 | FR681951 |
| Alc-0 | FR681983 | FR682060 | FR681952 |
| Blh-1 | FR681984 | FR682061 | FR681953 |
| Gre-0 | FR681985 | FR682062 | FR681954 |
| Mh-1 | FR681986 | FR682063 | FR681955 |
| Oy-0 | FR681987 | FR682064 | FR681956 |
| Shahdara | FR681988 | FR682065 | FR681957 |
| Akita | FR681989 | FR682066 | FR681958 |
| Sakata | FR681990 | FR682067 | FR681959 |
| N13 | FR681991 | FR682068 | FR681960 |
| Ws-2 | FR681992 | FR682069 | FR681961 |
| Ler-2 | FR681993 | FR682070 | FR681962 |
| C24 | FR681994 | FR682071 | FR681963 |
| Ge-1 | FR681995 | FR682072 | FR681964 |
| Ll-0 | FR681996 | FR682073 | FR681965 |
| Wu-0 | FR681997 | FR682074 | FR681966 |
| Nd-1 | FR681998 | FR682075 | FR681967 |
